# Physiologic Lesion Assessment to Optimize Multivessel Disease

**DOI:** 10.1007/s11886-022-01675-8

**Published:** 2022-03-02

**Authors:** Murtaza Bharmal, Morton J. Kern, Gautam Kumar, Arnold H. Seto

**Affiliations:** 1 University of California, Irvine, USA; 2 Veterans Administration Long Beach Health Care System, Long Beach, USA; 3 Atlanta Veterans Administration Medical Center, Atlanta, USA; 4 Emory University, Atlanta, USA

**Keywords:** Coronary hemodynamics, Coronary artery disease, Fractional flow reserve, Non-hyperemic pressure ratios, coronary artery bypass graft, acute coronary syndrome

## Abstract

**Purpose of Review:**

Multivessel coronary artery disease, defined as significant stenosis in two or more major coronary arteries, is associated with high morbidity and mortality. The diagnosis and treatment of multivessel disease have evolved in the PCI era from solely a visual estimation of ischemic risk to a functional evaluation during angiography. This review summarizes the evidence and discusses the commonly used methods of multivessel coronary artery stenosis physiologic assessment.

**Recent Findings:**

While FFR remains the gold standard in coronary physiologic assessment, several pressure-wire-based non-hyperemic indices of functional stenosis have been developed and validated as well as wire-free angiographically derived quantitative flow ratio. Identifying and treating functionally significant coronary atherosclerotic lesions reduce symptoms and major adverse cardiovascular events.

**Summary:**

Coronary physiologic assessment in multivessel disease minimizes the observer bias in visual estimates of stenosis, changes clinical management, and improves patient outcomes.

## Introduction

**Fig. 1:**
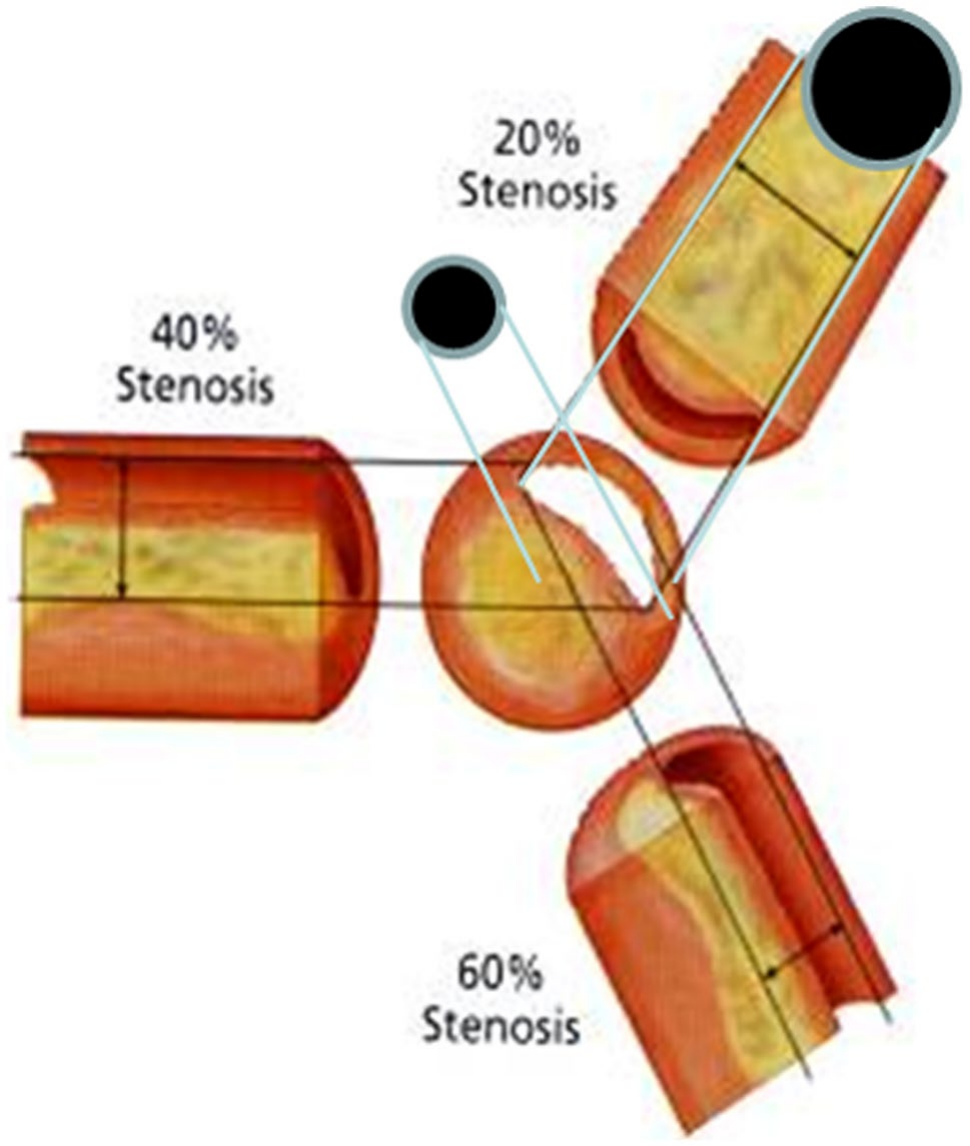
Eccentric lesions can create the appearance of 20–60% stenosis on angiography depending on the angle of inspection

Since its inception, coronary angiography has been a standard for the diagnosis and management of coronary artery disease. Multivessel coronary artery disease, defined as significant stenosis in two or more major coronary arteries, is associated with the highest morbidity and mortality. Significant lesions are denoted visually by 70% luminal diameter narrowing on angiography. However, visual stenosis assessment is plagued by interobserver variability and imprecision. Operators cannot accurately predict physiologic significance, especially for intermediate lesions in the range of 50 to 70% diameter stenosis (Fig. 1) [1, 2]. Moreover, treating functionally significant coronary atherosclerotic lesions reduces major adverse cardiovascular events (MACE) and symptoms [3–5]. Thus, the focus of this review is to summarize the evidence and commonly used methods for physiologic assessment of multivessel disease.

## Types of Physiologic Assessment

**Fig. 2:**
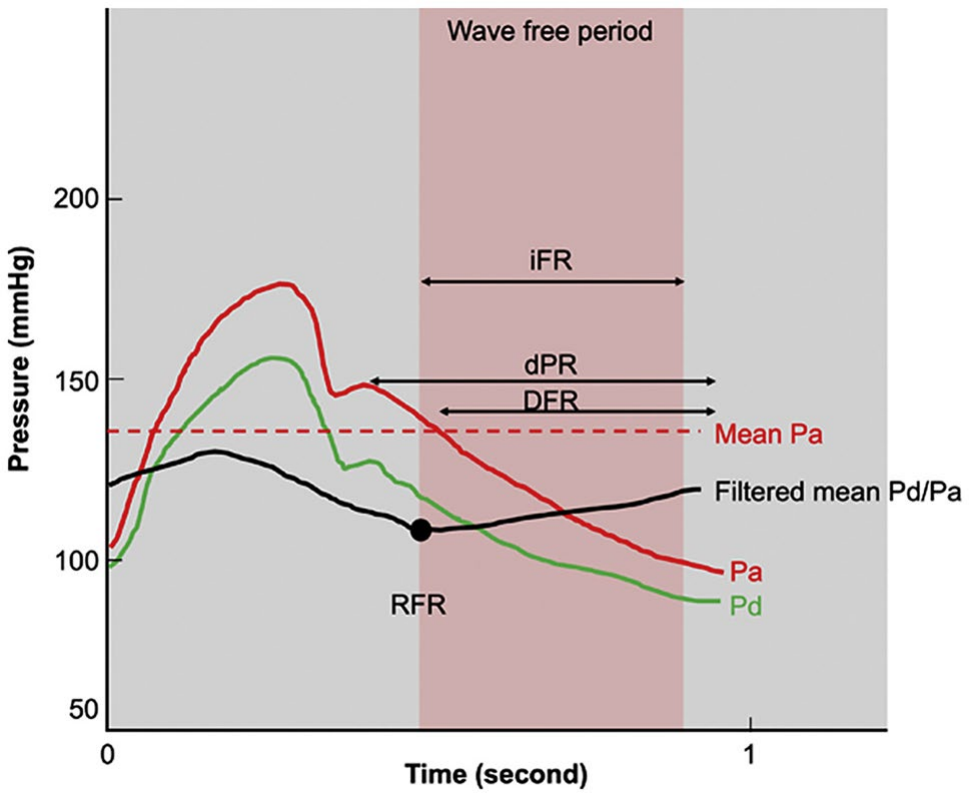
Non-hyperemic pressure ratio derivations. Instantaneous wave-free ratio (iFR) is defined as average Pd/Pa during the wave-free period (WFP) (*pink shaded area*). The WFP was calculated beginning 25% of the way into diastole and ending 5 ms before the end of diastole. Diastolic pressure ratio (dPR) is defined as average Pd/Pa during entire diastole. Diastolic hyperemia-free ratio (DFR) is defined as average Pd/Pa during Pa less than mean Pa with negative slope. Resting full-cycle ratio (RFR) is defined as the lowest filtered mean Pd/Pa during the entire cardiac cycle (adapted from Kogame et al. J Am Coll Cardiol Intv 2020;13:1617–1638, with permission from Elsevier) [54]

**Fig. 3:**
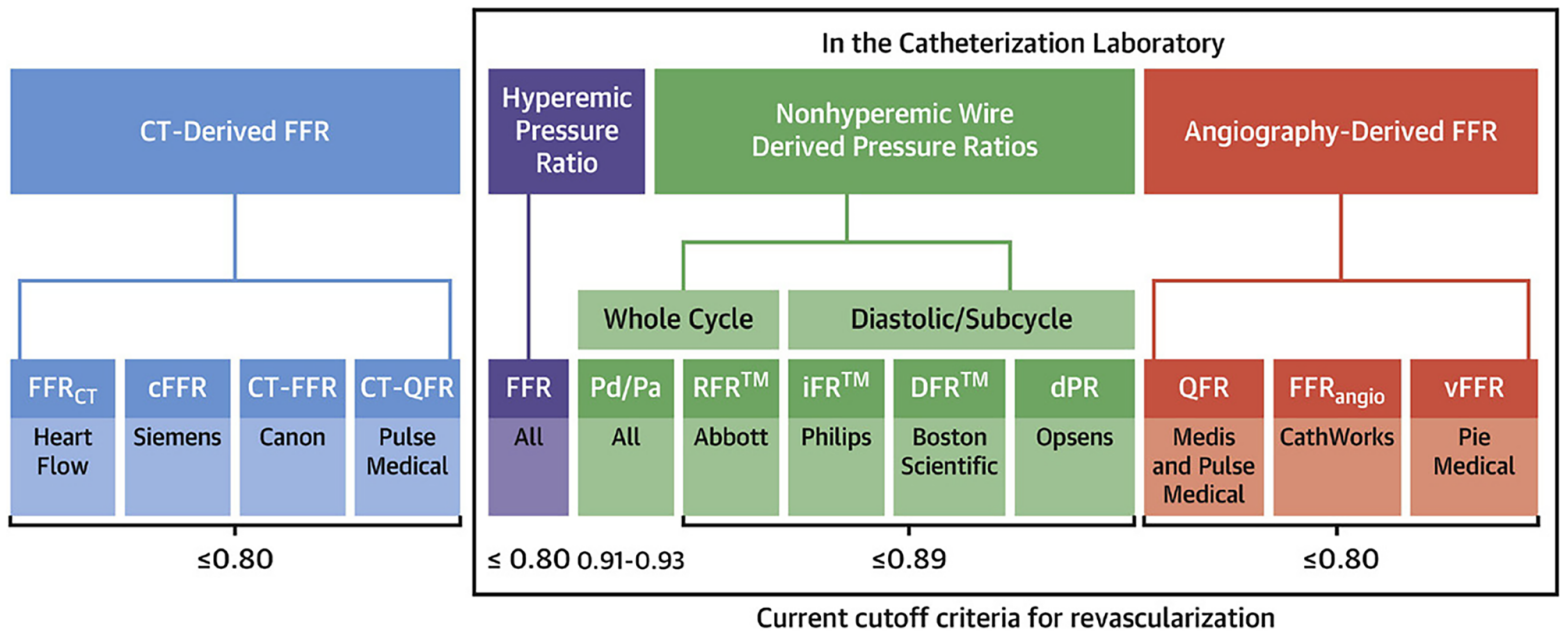
Currently available physiological assessment outside the cath lab (**left panel**) and in the catheterization laboratory (**right panels**) (from Kogame et al. J Am Coll Cardiol Intv 2020;13:1617–1638, with permission from Elsevier) [54]

Although fractional flow reserve (FFR) is the gold standard in invasive physiologic assessment of coronary stenosis, it remains underutilized due to several factors including operator reluctance to delay procedures, hospital costs, conceptual skepticism, and side effects of hyperemic pharmacologic agents. Non-hyperemic pressure ratios (NHPR) using the resting distal/aortic pressure over either the whole cardiac cycle or confined to diastole only were developed as an alternative to FFR (Fig. 2, Table 1). The simplest of the NHPRs is the resting pressure ratio of the mean distal/aortic pressure over the whole cardiac cycle, Pd/Pa, which has an 80–85% concordance with FFR using a threshold value of ≤ 0.91 [6]. Further refining the NHPR measurements, the first diastolic sub-cycle index, the instantaneous wave-free ratio (iFR), has been supported by large randomized clinical studies showing non-inferiority to FFR for physiology-guided revascularization [7]. As one might expect in comparison to FFR, omitting adenosine for routine iFR use results in reduced patient discomfort, time, and cost [8, 9]. Newer NHPR methodologies like resting full-cycle ratio (RFR™, Abbott), diastolic hyperemia-free ratio (DFR™, Boston Scientific), and diastolic pressure ratio (dPR™, Acist and Opsens Medical) have been found to be numerically identical to iFR and thus interchangeable for clinical use (Fig. 3) [10–12].

**Table 1:** Key indices of physiologic assessment for multivessel disease

Method	Ischemic threshold	Advantages	Limitations	Studies
Fractional flow reserve (FFR)	0.80	• Well-validated with non-invasive functional testing and 15-year outcomes data • Valid in non-culprit ACS vessel	• Hyperemia required with possible adverse pharmacologic effects • Pressure wire required • Prolonged procedure time	• FAME [14] and FAME II [15, 16••] • DANAMI-3-PRIMULTI [25] • COMPARE-ACUTE [26] • 3 V-FFR FRIENDS [22]
Instantaneous flow reserve (iFR™, Philips)	0.89	• Well-validated with non-invasive functional testing and 15-year outcomes data • Hyperemia independent • Pullback useful in serial and diffuse lesions • Angiography co-registration available	• Pressure wire required • Proprietary and specific software required	• iFR-SWEDEHEART [9] • DEFINE FLAIR [8] • SYNTAX II [17] • Maini et al. [7]
Resting full-cycle ratio (RFR™, Abbott) Diastolic hyperemia-free ratio (DFR™, Boston Scientific) Diastolic pressure ratio (dPR, Acist and Opsens Medical)	0.89	• Hyperemia independent • Good correlation with FFR/iFR	• No outcomes data available • Pressure wire required • Proprietary and specific software required	• VALIDATE RFR [10] • Johnson et al. [11] • VERIFY2 [12]
Distal to aortic pressure ratio (Pd/Pa)	0.91	• Hyperemia independent • Good correlation with iFR	• No outcomes data available • Pressure wire required • Low fidelity for serial stenosis assessment	• Kobayashi et al. [6]
Quantitative flow reserve (QFR)	0.80	• Well-validated against FFR • Hyperemia independent • No pressure wire required • Instantaneous FFR computation • Flow estimated from patient-specific data and TIMI frame count	• No outcomes data available • Precise angiography images required • Specific software required • Nitroglycerin administration required	• FAVOR Pilot [44], FAVOR II China [46] • WIFI II [45] • PANDA III [47] • Spitaleri et al. [48]
Fractional flow reserve by angiography (FFR_angio_)	0.80	• Hyperemia independent • No pressure wire required • Computation time < 5 min • Complete coronary tree functional assessment	• No outcomes data available • Precise angiography images required • Specific software required	• FAST-FFR [50•]
Virtual fractional flow reserve (vFFR)	0.80	• Hyperemia independent • No pressure wire required • Computation time < 5 min	• No outcomes data available • Rotational angiography required • Specific software required	• FAST II [51]
Computed tomography fractional flow reserve (CT-FFR)	0.80	• Non-invasive and no pressure wire required • Hyperemia independent • Combination of functional and anatomic data	• No outcomes data available • Proprietary and specific software required • Need for supercomputer computations limits availability	• SYNTAX III Revolution [41]

*ACS* acute coronary syndrome, *TIMI* thrombolysis in myocardial infarction

## Rationale for Physiologic Assessment of Coronary Stenoses

Given that myocardial ischemia is an important prognostic factor for patients with coronary artery disease [13], defining ischemia-causing stenosis is critical, particularly in patients with multivessel disease. However, because of the complexity of anatomy and overlap of ischemic territories and other factors, angiographic and nuclear stress imaging assessment in patients with multivessel disease often cannot discern which lesions are important nor identify the degree of myocardial ischemia present. Defining ischemia-causing stenosis not only changes patient management strategies but also impacts clinical outcomes.

Improved outcomes have been associated with physiological assessment for multivessel coronary artery disease (Table 2). For example, in a 5-year follow-up to the FAME (Fractional Flow Reserve Versus Angiography for Multivessel Evaluation) study, major adverse cardiovascular events in the FFR-guided and angiography-guided PCI groups were similar (28% vs 31%, relative risk 0.91, 95% CI 0.75–1.10; *p* = 0.31) despite a higher number of stents placed in the angiography group, suggesting that a similar outcome can be achieved with fewer stents used selectively for lesions of hemodynamic significance [14]. The FAME 2 trial aimed to clarify if FFR-guided PCI in addition to optimal medical therapy is superior to optimal medical therapy alone for patients with stable coronary artery disease, with multivessel disease consisting of 26% and 22% of patients, respectively. [15] Not only was the trial stopped prematurely due to a significantly lower rate of death, myocardial infarction, or urgent revascularization in the FFR-guided PCI group of the study at 2 years but these results were sustained at 5 years. [16••] Further evidence of improved outcomes was observed in the SYNTAX II trial, which was a comprehensive strategy of three-vessel disease management using the SYNTAX Score II, iFR/FFR-guided revascularization, and intravascular ultrasound-guided implantation. Results showed reduced major adverse cardiac and cerebrovascular events compared to PCI performed in comparable patients from the SYNTAX I trial (HR 0.58, 95% CI 0.39–0.85, *P* = 0.006) [17].

**Table 2:** Key outcomes studies evaluating coronary physiology in multivessel disease

Study	Topic	Design	Method	No. of patients	Primary endpoint	Outcome
DANAMI-3-PRIMULTI [25]	CR vs COR in STEMI pop	RCT	FFR	627	Composite (all-cause death, non-fatal MI, ischemia-driven TLR)	Complete: 13% Culprit: 22% *P* = 0.004
COMPARE-ACUTE [26]	CR vs COR in STEMI pop	RCT	FFR	885	Composite (all-cause death, non-fatal MI, revascularization, stroke)	Complete: 8% Culprit: 21% *P* < 0.001
COMPLETE [28]	CR vs COR in STEMI pop	RCT	FFR (< 1%)	4041	Composite (cardiovascular death, non-fatal MI)	Complete: 7.8% Culprit: 10.5% *P* = 0.004
FLOWER-MI [29]	CR vs COR in STEMI pop	RCT	FFR	1171	Composite (all-cause death, non-fatal MI, urgent revascularization)	Angio complete: 4.2% FFR complete: 5.5% *P* = 0.31
FULL-REVASC [30•]	CR vs COR in STEMI pop	RCT	FFR	4052*	Composite (all-cause death, non-fatal MI)	-
iMODERN (2021) [31]	CR vs COR in STEMI pop	RCT	iFR	1146*	Composite (all-cause death, recurrent MI, heart failure hospitalization)	-
FAME – 5 Year Follow-Up [14]	Stable MVD	RCT	FFR	1005	Composite (all-cause death, MI, urgent revascularization) 5-year outcomes	Angio: 31% FFR: 28% *P* = 0.31
FAME 2 – 5 Year Follow-Up [16••]	Stable MVD	RCT	FFR	888	Composite (all-cause death, MI, urgent revascularization)	PCI: 13.9% Medical: 27.0% *P* < 0.001
DEFINE-FLAIR [8]	Stable CAD	RCT	iFR vs FFR	2492	Composite (all-cause death, non-fatal MI, revascularization)	FFR: 7% iFR: 6.8% *P* < 0.001
SYNTAX II [17]	Stable MVD	Single arm study	FFR/iFR	708	Composite (all-cause death, stroke, any MI, any revascularization)	SYNTAX II: 10.6% SYNTAX I: 17.4% *P* = 0.006
3 V-FFR FRIENDS [22]	Stable MVD	Prospective cohort	FFR	1136	Composite (all-cause death, MI, revascularization)	Low 3 V FFR (< 2.72): 7.1% High 3 V FFR (≥ 2.72): 3.8% *P* = 0.011

*CR* complete revascularization, *COR* culprit-only revascularization, *STEMI* ST elevation myocardial infarction, *RCT* randomized controlled trial, *TLR* target lesion revascularization, *MVD* multivessel disease, *CAD* coronary artery disease

^*^Estimated enrollment

Based on physiologic assessment, changes in patient management (medical vs PCI vs CABG) may explain the improvement in patient outcomes. Van Belle et al. assessed the impact of routine invasive physiology at the time of angiography on decision reclassification in patients with multivessel disease [18•]. Investigators were asked to define their management strategy based on angiography and clinical information alone, perform invasive physiologic assessment by FFR or iFR, and define their final treatment strategy after physiologic assessment. Reclassification occurred in 30% of patients, ultimately leading to different patient management in 26.9%. In particular, as the number of vessels investigated increased, reclassification of patient care increased accordingly. Similarly, Ahn et al. noted an 11% absolute reduction in referral to CABG after routine FFR use [19]. The risk of major adverse cardiovascular and cerebral events was reduced by 43% compared to prior non-routine FFR use. Moreover, the risk of MACE was higher in PCI patients compared to CABG patients prior to the routine use of FFR (hazard ratio [HR] 1.82, 95% confidence interval [CI] 1.09 to 3.03, *p* = 0.021), and was not significantly different after routine incorporation of FFR (HR 1.22, 95% CI 0.59 to 2.52, *p* = 0.59). These results, however, were not reflected in the FAME 3 trial where FFR-guided PCI did not meet non-inferiority compared to CABG among patients with multivessel disease at 1 year follow-up [20]. Subgroup analysis did suggest that patients with a low SYNTAX score of 0–22 appeared to be a benefit from PCI versus CABG; however, further studies are needed to confirm these findings.

Lesions with borderline FFR carry an unknown ischemia risk. Park et al. identified patients with multivessel CAD having lesions with borderline FFR values (FFR 0.81–0.87) and this cohort was found to have a comparable risk of MACE at 2 years compared to patients with functionally significant CAD, defined by FFR ≤ 0.80 (HR 1.2, 95% CI 0.5–3.0%; *P* = 0.67) [21•]. Identifying these higher risk patients for adverse outcomes may allow for more aggressive medical therapy and surveillance.

In the 3 V-FFR FRIENDS (3-Vessel Fractional Flow Reserve for the Assessment of Total Stenosis Burden and Its Clinical Impact in Patients with Coronary Artery Disease) study, Lee and colleagues routinely examined 3-vessel FFR measurements (i.e., summed FFR of three major vessels) in patients with multivessel disease to understand the influence of total atherosclerotic burden on clinical outcomes. Patients with high total physiologic atherosclerotic burden (defined as a low 3 V-FFR value < 2.72) were associated with a higher MACE at 2 years compared to low total physiologic atherosclerotic burden (defined as a high 3 V-FFR value ≥ 2.72), which was mainly a result of higher rate of ischemia-driven revascularization [22]. In a subset of patients identified to have insignificant angiographic stenosis (percentage of diameter stenosis < 50% by visual assessment) with deferred intervention (8.7% of study group), those patients with lesions having a low FFR (≤ 0.80) showed a significantly higher risk of MACE compared to patients with a high FFR (> 0.80) at 2 years (3.3% versus 1.2%, hazard ratio: 3.371; 95% CI, 1.346–8.442; *P* = 0.009) [23]. Low FFR and iFR were both associated with future risk of MACE in deferred lesions [24].

## STEMI and Non-culprit Physiologic Assessment

For patients with STEMI and multivessel disease, a commonly raised question is whether revascularization should be directed at the culprit only or at all significant stenoses during or soon after the index procedure. Most studies evaluating complete revascularization in STEMI patients used visualization estimates from coronary angiography regarding lesion severity for decision-making. The first two trials to incorporate routine FFR-guided revascularization were the DANAMI-3-PRIMULTI and COMPARE-ACUTE trials. In the DANAMI-3-PRIMULTI trial, patients presenting with STEMI who had more than one visually significant stenosis in the non-infarct-related artery underwent randomization to no further therapy or FFR-guided completed revascularization during index hospitalization. While there was a significant reduction in future revascularization procedures, there was no difference in all-cause mortality or non-fatal myocardial infarciton [25]. The COMPARE-ACUTE trial (Fractional Flow Reserve-Guided Multivessel Angioplasty in Myocardial Infarction) found a lower risk of MACE or cerebrovascular events in the FFR-guided group driven primarily by a lower risk of revascularization [26], reaching the same conclusion as the prior trial. After the release of this study’s results in 2017, the European Society of Cardiology guidelines subsequently recommended that PCI for non-culprit lesions should be considered (class IIa) for the treatment of patients with STEMI and multivessel disease [27]. Subsequent evaluation in the COMPELTE trial demonstrated consistent results with previous trials of reduced risk of cardiovascular death or myocardial infarction, granted that physiologic lesion assessment was limited to intermediate lesions which consisted of < 1% of non-culprit lesion stenosis [28]. With these results, FFR-guided complete revascularization may not necessarily improve death and myocardial infarction but likely reduce repeat revascularization, suggesting indirect beneficial effects on quality of life and healthcare cost-utility.

Contrary to the aforementioned trials, the FLOWER-MI (Flow Evaluation to Guide Revascularization in Multivessel ST Elevation Myocardial Infarction) trial suggested that a FFR-guided strategy did not significantly reduce outcomes of death, MI, or urgent revascularization when compared to angiography-guided revascularization strategy. [29] However, the observed event rates in both arms of the study were much lower than expected, underpowering the study, resulting in a wide confidence interval, and leading to inconclusive results.

Given the conflicting results of studies in this specific population, questions regarding the true value of FFR in the acute coronary syndrome population have arisen. The FFR guidance for complete non-culprit revascularization (Full-Revasc) trial is a large, multi-center, randomized clinical trial designed to evaluate the effect of FFR-guided complete revascularization of non-culprit lesions during index hospitalization on total mortality, non-fatal MI, and unplanned revascularization. Enrollment completed in September 2019 and the follow-up is still ongoing [30•]. Additionally, the iFR-guided multivessel revascularization during percutaneous coronary intervention for acute myocardial infarction (iMODERN) trial is ongoing and will compare an iFR-guided intervention of non-culprit lesions during acute intervention with a deferred stress perfusion CMR-guided strategy [31].

## Diffuse Disease and Serial Lesion Physiologic Assessment

**Fig. 4:**
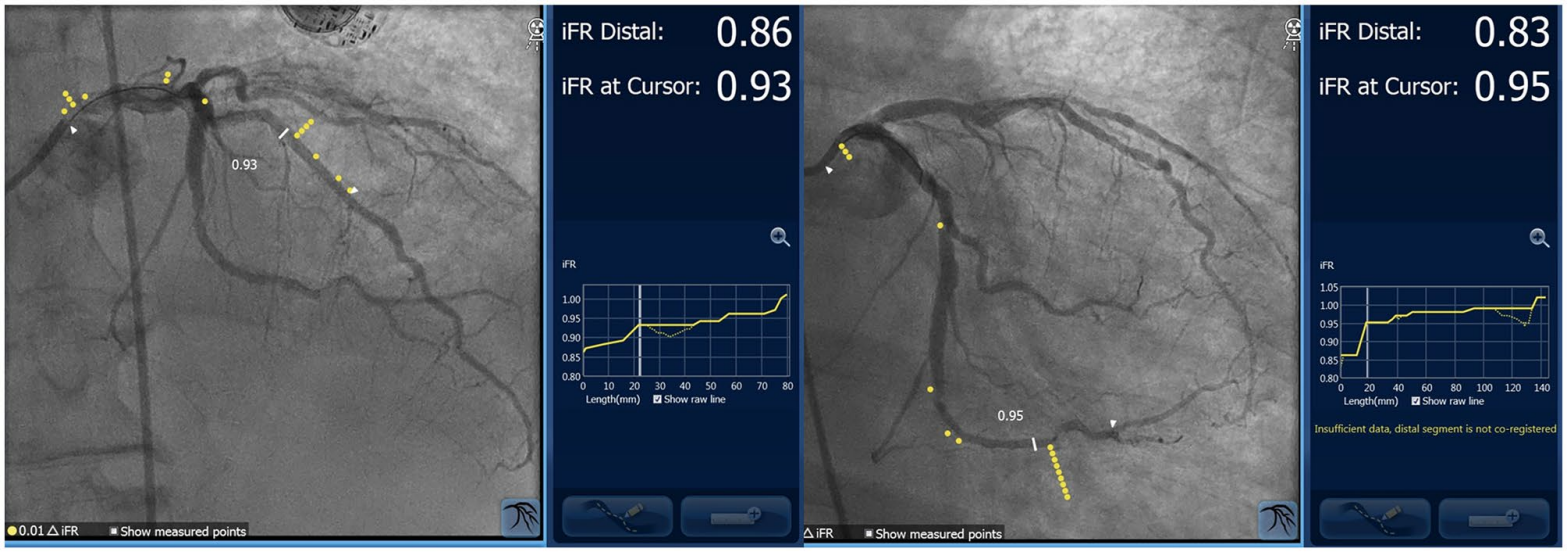
Pull back pressure recordings. (**Left**), diffuse disease. (**Right**), focal step up in distal vessel with no gradient across circumflex ostium

Advanced atherosclerotic coronary disease often presents as diffuse and/or serial lesions which complicates simple physiologic assessment. In serial or tandem lesions, individual stenosis FFR cannot be determined because of lesion interaction and limited maximal lesion hyperemia. The contribution of each lesion to the total resistance during maximal hyperemia can be challenging to deconstruct and measure by FFR. In contrast, NHPRs, like iFR, do not require hyperemia and theoretically do not have as much lesion interaction. Performing a pressure-wire pullback study can superimpose iFR on the angiogram to create a blueprint of functionally significant coronary stenosis, differentiating diffuse from focal disease (Fig. 4). For diffuse disease, the distributed pressure gradient may be gradual along the entire vessel whereas focal areas of sharp pressure change identify the most physiologically discrete lesions. Stent placement in these specific areas may maximize post-PCI blood flow with the appropriate amount of stent usage. Compared with angiography alone, iFR pullback altered revascularization procedural planning in 31% of patients [32••, 33]. In addition, iFR pullback recordings that identified predominantly physiologically focal versus diffuse disease was an important factor for FFR/iFR discordance, with focal disease associated with FFR + /iFR- and diffuse disease associated with FFR-/iFR + results [34•]. Hyperemic pressure pullback gradient, a metric of pressure drop over 20 mm measured by a pressure-wire pullback device with a set speed of 1 mm/s, can also characterize pathophysiological patterns of focal versus diffuse disease with similar results [35].

## Post-PCI Physiology

While pre-PCI physiology has become routine for functional lesion assessment, physiologic measurements are only occasionally performed post-PCI in clinical practice, despite the presence of residual ischemia in up to 24% of patients [36]. In a similar fashion, recent data suggest that a greater physiological improvement in lesion stenosis corresponds to greater symptomatic relief and a lower event rate [37]. In patients who received functionally complete revascularization (CR), instead of angiographic-based revascularization, lower post-PCI FFR was associated with an increased risk of target vessel failure (hazard ratio, 1.091 [95% CI, 1.032–1.153]; *P* = 0.002) [38]. Likewise, patients who received functionally incomplete revascularization (IR) showed a higher incidence of MACE at 2 years than those with functionally complete revascularization (functional IR vs CR, 14.6% vs 4.2%; hazard ratio: 4.09; 95% confidence interval: 1.82 to 9.21; *p* < 0.001) [39].

## Future Directions

Future directions in coronary physiologic assessment of multivessel disease will likely incorporate regular use of imaging modalities like CT-FFR and angiographic-derived FFR such as quantitative flow ratio (QFR) among others. In the SYNTAX III Revolution trial, a comprehensive heart team decision on optimal medical therapy (CABG, PCI, or equipoise between CABG and PCI) by coronary CT-FFR for multivessel disease showed a high agreement with the decision by conventional coronary angiography, allowing for adequate decision-making and procedural planning in up to 20% of patients on non-invasive physiologic assessment alone [40–42].

**Fig. 5:**
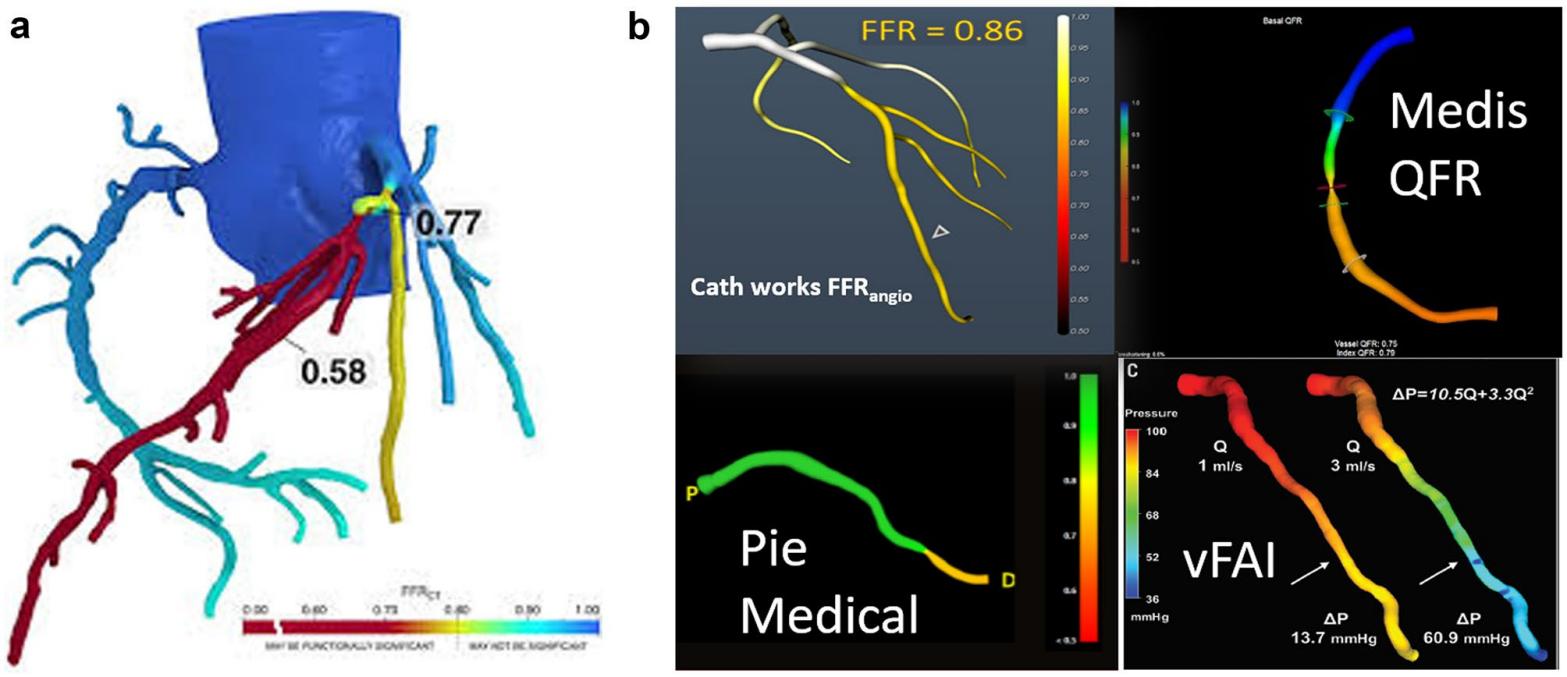
(**A**) Methodologies for angiographic-derived FFR include CT-based FFR (HeartFlow) prior to invasive physiology, while (**B**) in-lab approaches include QFR (Medis), FFR_angio_ (CathWorks), CAAS-vFFR (Pie Medical), and vFAI (virtual functional assessment index)

Despite the availability and accuracy of pressure-wire-based hyperemic and non-hyperemic measures of coronary physiology, their routine use remains limited. Functional coronary stenoses assessment by wire-free angiographically derived 3D reconstruction and computational flow dynamic calculations has led to the development of angiographic FFR such as QFR (Fig. 5). Two high-quality angiographic projections are used to reconstruct a 3D coronary tree and frame count analysis allows for computation of simulation models (fixed flow, contrast flow, and adenosine flow) to identify functionally significant stenosis [43]. Several studies have validated the accuracy and high correlation of QFR with FFR as a diagnostic reference [44–46]. Furthermore, QFR incorporation into functional SYNTAX score improves the prognostication and revascularization strategy choice compared to anatomic assessment alone in patients with left main or multivessel coronary artery disease [47]. QFR computation may also be a reliable tool to guide revascularization of non-infarct coronary arteries in the acute setting for patients with STEMI [48, 49]. Other indices of functional assessment including FFR derived by angiography along (FFR_angio_) and three-dimensional quantitative coronary angiography (3D-QCA)–based FFR (vFFR) also show high accuracy when compared to pressure-wire-based FFR in diagnosing functionally significant stenosis [50•, 51]. Finally, the interpretation of wire-based physiologic measurements can be made more accurate and reproducible with the use of artificial intelligence, which may ultimately be applied for decision-making irrespective of the data source [52, 53].

## Conclusions

Physiologic assessment of multivessel coronary artery disease minimizes the observer bias in visual estimates of stenosis, particularly for intermediate severity stenosis. Strong clinical evidence and guideline recommendations support routine use of physiologic assessment for more accurate identification and treatment for ischemia-related stenosis. Building on FFR, novel techniques to assess physiology with non-hyperemic indices like iFR and angiographically derived FFR have a growing body of evidence for routine use. A physiologic-guided approach will likely provide optimal revascularization strategies, objectively assess the adequacy of post-intervention, and will be associated with improved outcomes. As the techniques and evidence for physiologic coronary assessment continue to grow, these tools will undoubtedly shift the focus of contemporary treatment from anatomic to functional revascularization in patients with multivessel coronary disease.
