# Correction to: Physiologic Lesion Assessment to Optimize Multivessel Disease

**DOI:** 10.1007/s11886-022-01696-3

**Published:** 2022-04-08

**Authors:** Murtaza Bharmal, Morton J. Kern, Gautam Kumar, Arnold H. Seto

**Affiliations:** 1 University of California, Irvine, USA; 2 Veterans Administration Long Beach Health Care System, Long Beach, USA; 3 Atlanta Veterans Administration Medical Center, Atlanta, USA; 4 Emory University, Atlanta, USA

## Correction to: Current Cardiology Reports https://doi.org/10.1007/s11886-022-01675-8

The article “Physiologic Lesion Assessment to Optimize Multivessel Disease,” written by Bharmal, M., Kern, M.J., Kumar, G., and Seto, A.H., was originally published electronically on the publisher’s internet portal on 02 March 2021 with error on Figs. 2 and 5 captions.

In Fig. 2, right and left panel references should be removed since there is only one panel.

In Fig. 3 panel A, the word “angiography” should be replaced with “physiology.”

The original article has been corrected.

